# Belantamab mafodotin, pomalidomide and dexamethasone in refractory multiple myeloma: a phase 1/2 trial

**DOI:** 10.1038/s41591-023-02703-y

**Published:** 2024-01-04

**Authors:** Suzanne Trudel, Arleigh McCurdy, Martha L. Louzada, Stephen Parkin, Darrell White, Michael P. Chu, Rami Kotb, Hira Mian, Ibraheem Othman, Jiandong Su, Aniba Khan, Engin Gul, Donna Reece

**Affiliations:** 1 Princess Margaret Cancer Centre, University Health Network, Toronto, Ontario Canada; 2 Ottawa Hospital Research Institute, Ottawa, Ontario Canada; 3 London Health Sciences Centre, London, Ontario Canada; 4 Vancouver General Hospital, Vancouver, British Columbia Canada; 5 Queen Elizabeth II Health Sciences Centre, Dalhousie University, Halifax, Nova Scotia Canada; 6 Cross Cancer Institute, Edmonton, Alberta Canada; 7 CancerCare Manitoba, Winnipeg, Manitoba Canada; 8 Juravinski Cancer Centre, McMaster University, Hamilton, Ontario Canada; 9 Allan Blair Cancer Centre, Regina, Saskatchewan Canada; 10 Canadian Myeloma Research Group (CMRG), Vaughan, Ontario Canada

**Keywords:** Myeloma, Education

## Abstract

Due to evolving treatment standards for newly diagnosed multiple myeloma, many patients will be triple-class exposed after initial relapses and have poor survival. Novel therapies and combinations are therefore required to improve outcomes. B cell maturation antigen (BCMA)-targeted biologics have emerged as an important new area of therapeutics for relapsed multiple myeloma. The two-part ALGONQUIN trial evaluated various doses and schedules of the anti-BCMA antibody–drug conjugate belantamab mafodotin plus pomalidomide and dexamethasone for patients who are lenalidomide refractory and proteosome inhibitor exposed. The primary endpoints, including evaluating dose-limiting toxicities, establishing the recommended Part 2 dose (RP2D) and overall response rate for patients treated at the RP2D, were met. Secondary efficacy endpoints included progression-free survival and overall survival. Patients treated on study (*N* = 87) had a median of three previous regimens and 55.2% were triple-class refractory. At the RP2D the most common adverse events were decrease in best-corrected visual acuity (71.1%), keratopathy (65.8%), fatigue (57.9%), infection (47.4%; 7.9% grade ≥3), neutropenia (39.5%) and thrombocytopenia (39.5%). For RP2D patients (*n* = 38), the overall response rate was 85.3%, ≥very good partial response 75.7% and estimated two-year progression-free survival 52.8% (95% confidence interval, 33.9% to 82.4%), at a median follow-up of 13.9 months. The RP2D schedule was associated with manageable antibody–drug conjugate-associated corneal adverse events and improved tolerability without compromising efficacy. Belantamab mafodotin plus pomalidomide and dexamethasone induced durable responses with promising overall survival in relapsed multiple myeloma, the results of which are yet to be confirmed in the phase 3 DREAMM-8 study. ClinicalTrials.gov Identifier: NCT03715478.

## Abstract (web summary)

Treatment of patients with relapsed or refractory multiple myeloma with a combination of the BCMA-targeting antibody–drug conjugate belatamab mafodotin with dexamethasone and pomlidomide is safe and shows promising preliminary clinical efficacy.

## Main

Multiple myeloma (MM) is a plasma cell malignancy expressing B cell maturation antigen (BCMA). Treatment is evolving rapidly, with new therapeutics leading to notable improvements in survival. Despite this, relapse is expected, and MM remains incurable in most patients. Current treatments for patients with newly diagnosed or early relapsed MM include various combinations of proteosome inhibitors (PIs), immunomodulatory drugs (IMiDs) and monoclonal antibodies (mAbs) targeting CD38, as well as autologous stem cell transplantation in select fit patients. At the time of relapse, additional novel agents and combinations are required to control disease and improve survival.

Belantamab mafodotin (belamaf) is an afucosylated humanized anti-BCMA IgG1 mAb conjugated to microtubule disrupting monomethyl auristatin F (MMAF). The rapid internalization of the MMAF cytotoxic payload induces immune-independent apoptosis in addition to immunogenic cell death. Furthermore, binding to FcγRIIIa on plasma cells results in the activation and recruitment of immune effector cells and enhanced antibody-dependent cell-mediated cytotoxicity^1^. The multimodal activity of belamaf differentiates it from currently approved BCMA-targeting agents for patients with relapsed and/or refractory MM (RRMM). In the phase 2 DREAMM-2 trial, of single-agent belamaf (2.5 mg kg^−1^ every 3 weeks) in patients with RRMM refractory to a PI, an IMiD and an anti-CD38 mAb, the overall response rate (ORR) was 31%, with median duration of response of 12.5 months (ref. ^2^). Despite confirmation of the single-agent activity of belamaf in the randomized DREAMM-3 study, with an ORR of 41% and median progression-free survival (mPFS) of 11.2 months, these endpoints were not statistically superior to the comparator arm of pomalidomide and dexamethasone (Pd). It is notable, however, that the median duration of response (mDOR) was 25.6 months with 10 additional months of follow-up. The encouraging durability of responses observed with single-agent belamaf supported its further development but the data from DREAMM-3 suggest that combination strategies are likely required.

Pd is an established standard therapy in relapsed MM, with original approval based on the randomized MM-003 trial showing an ORR of 31% and median mPFS of 4.0 months (ref. ^3^), leaving room for improvement with combinations. In addition to direct cytotoxic anti-MM effects^4^, pomalidomide augments natural killer and T cell-mediated immunity and enhances antibody-dependent cell-mediated cytotoxic activity^5–8^. Multiple studies combining Pd with mAbs for the treatment of relapsed MM have shown significantly improved clinical efficacy and a manageable safety profile^9–12^, supporting the use of Pd in combination with belamaf in the present trial.

Given that most current patients will have been exposed to anti-CD38 mAbs in first- or second-line therapy, exploration of Pd combinations with agents exploiting novel targets is needed. The ALGONQUIN trial is a multicenter, single-arm, open-label, dose-exploration two-part study designed to evaluate the safety and efficacy of belamaf in combination with Pd for the treatment of RRMM. Herein, we report the results of the dose-exploration and dose-expansion portions of the trial.

## Results

### Patient disposition and baseline characteristics

Baseline patient characteristics are presented in Table 1. Between 4 January 2019 and 17 May 2022, 87 patients were enrolled and treated, including 61 in the Part 1 dose-exploration phase and 26 in the Part 2 dose-expansion phase. A total of 38 patients were treated at the RP2D (12 in Part 1 and 26 in Part 2). Across all cohorts the median age was 67 yr (range, 36–85), median time from diagnosis was 5 yr (range, 1–21) and median previous lines of therapy was 3 (range, 1–6). Nineteen of 71 (27%) patients had International Staging System stage 3 disease, and 16 of 45 (35.6%) with available fluorescence in situ hybridization data had high-risk cytogenetics (del(17p) and/or translocation t(4;14) and/or translocation t(14;16)). All patients were lenalidomide and PI exposed, 96.6% were lenalidomide refractory, 75 (86.2%) were refractory to lenalidomide and a PI, 58 (66.7%) had received previous anti-CD38 mAb therapy and 48 (55.2%) were triple-class refractory. Baseline patient characteristics for Part 1 cohorts are available in Extended Data Table 1.

**Table 1:** Baseline characteristics

Characteristics	Part 1 patients	RP2D patients	All patients
	*n* = 61	*n* = 38	*N* = 87^a^
Median age (range), yr	64 (36–81)	71 (38–85)	67 (36–85)
Female sex, n (%)	31 (50.8)	15 (39.5)	41 (47.1)
Median time since initial diagnosis (range), yr	5 (1–15)	4 (1–21)	5 (1–21)
Eastern Cooporative Oncology Group performance status			
0	20 (32.8)	10 (26.3)	25 (28.7)
1	35 (57.4)	26 (68.3)	55 (63.2)
2	6 (9.8)	1 (2.7)	6 (6.9)
Missing	0 (0.0)	1 (2.7)	1 (1.2)
Derived International Staging System stage at baseline, n (%)			
1	20 (16.3)	7 (18.4)	22 (25)
2	22 (36.1)	10 (26.3)	30 (34.4)
3	10 (16.4)	12 (31.6)	19 (21.8)
Missing	9 (14.8)	9 (23.7)	16 (18.4)
Baseline cytogenetics, n (%)			
High risk^b^	14 (23.0)	7 (18.5)	16 (18.4)
Standard risk^c^	18 (29.5)	14 (36.8)	29 (33.3)
Missing	29 (47.5)	17 (44.7)	42 (48.3)
Median no. of previous therapies (range)	3 (1–5)	3 (1–6)	3 (1–6)
Previous therapies, n (%)			
Autologous stem cell transplantation	49 (80.3)	18 (47.4)	60 (69.0)
Lenalidomide	61 (100.0)	38 (100.0)	87 (100.0)
PI	61 (100.0)	38 (100.0)	87 (100.0)
Anti-CD38	36 (59.0)	30 (78.9)	58 (66.7)
Triple-class exposure	36 (59.0)	30 (78.9)	58 (66.7)
Refractory to, n (%)			
Lenalidomide	58 (95.1)	36 (94.7)	84 (96.6)
PI	53 (86.9)	32 (84.2)	75 (86.2)
Anti-CD38	36 (59.0)	30 (78.9)	58 (66.7)
Triple-class refractory, n (%)	30 (49.2)	24 (63.2)	48 (55.2)

^a^Total of 87 patients from Part 1 (all cohorts) and Part 2.

^b^High risk is defined as patients presenting with abnormality for del(17p) and/or translocations t(4;14) and/or t(14;16). Fluorescence in situ hybridization was performed locally using the individual laboratory’s cut-off values.

^c^Standard risk is defined as patients with absence of abnormality for all of the following: del(17p), translocations t(4;14) and t(14;16).

**Extended Data Fig. 1:**
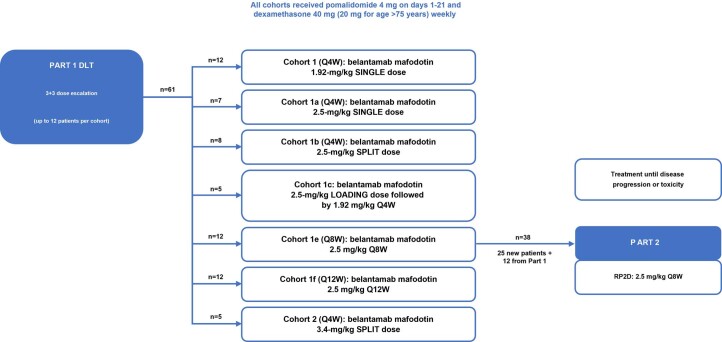
Study design for both the dose exploration and the RP2D parts of the study. DLT, dose-limiting toxicity; RP2D, recommended Part 2 dose.

Dose-exploration patients include patients from cohorts 1, 1a, 1b, 1c, 1e, 1f and 2 (Extended Data Fig. 1).

RP2D includes 12 patients from Part 1 cohort 1e and 26 patients from Part 2 (Extended Data Fig. 1).

**Fig. 1:**
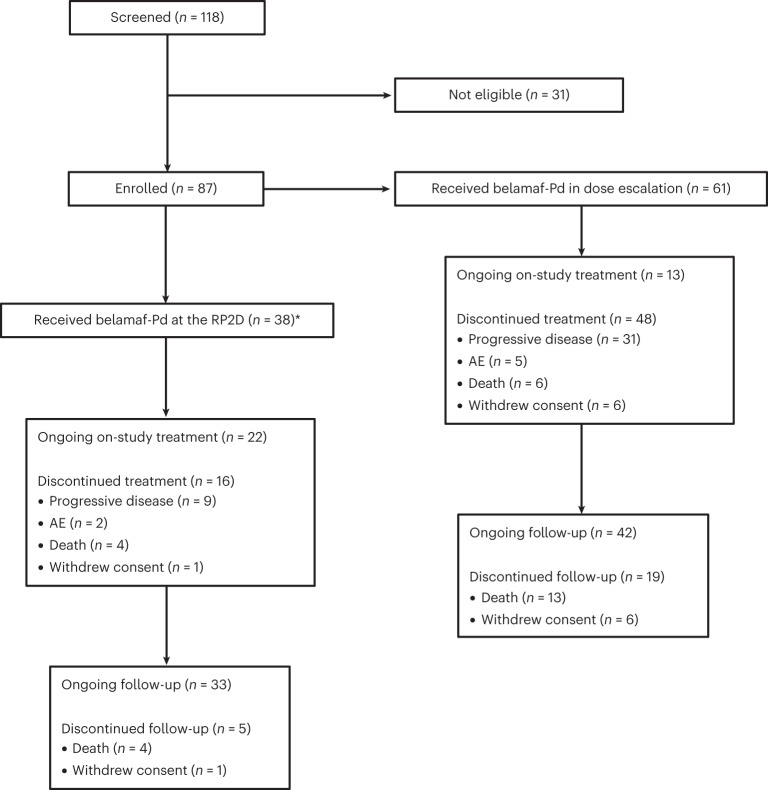
CONSORT diagram of the Algonquin study. Belamaf-Pd, belamaf, pomalidomide and dexamethasone. *12 patients from Part 1 and 26 patients from Part 2.

Patient disposition is provided in Fig. 1. At the time of data cut-off (14 February 2023), patients treated at the RP2D of belamaf 2.5 mg kg^−1^ every 8 weeks (Q8W) with Pd had a median duration of follow-up of 13.9 months (range, 1.1–28.2). Twenty-two patients (57.9%) were still receiving study treatment. The most common reason for discontinuation was disease progression in 9 of 38 (23.7%), while 2 of 38 (5.2%) discontinued treatment due to adverse events (AEs).

### Results of the dose-exploration cohorts

In Part 1, doses of 1.92, 2.5 and 3.4 mg kg^−1^ belamaf were administered according to various dosing schedules (Extended Data Fig. 1). Reasons for discontinuation are shown in Fig. 1. At a median follow-up of 17.1 months (range, 0.9–42.5), there were 12 of 61 (19.7%) deaths in Part 1, with 6 attributable to disease progression, 3 to COVID-19, 1 to pneumocystis pneumonia, 1 to influenza and 1 to myocardial infarction not related to study treatment. Five patients in Part 1 discontinued treatment due to an AE, with 2 reported as myelodysplastic syndrome possibly related to pomalidomide, 1 due to progressive multifocal leukoencephalopathy also attributed to pomalidomide, 1 secondary to increase in alanine transaminase possibly related to belamaf and 1 due to grade 4 decrease in best corrected visual acuity (BCVA) definitely related to belamaf. Two of 5 patients in the 3.4 mg kg^−1^ split (SPLIT) and 1 of 7 in the 2.5 mg kg^−1^ every 4 weeks (Q4W) cohorts experienced dose-limiting toxicities. All were due to grade 3 keratopathy, specifically 1 associated with grade 3 decreased BCVA and another reported with grade 4 decreased BCVA in the 3.4 mg kg^−1^ SPLIT dosing cohort. The maximum tolerated dose (MTD) based on the first cycle (28 d) of treatment was determined to be 2.5 mg kg^−1^ belamaf with Pd. The common treatment-emergent AEs (≥20%) and grade 3–4 AEs (≥5%) are shown in Table 2a,b and by cohort in Extended Data Table 2a,b. Consistent with previous reports for other MMAF-containing antibody–drug conjugates^13^, the most commonly reported AE was keratopathy found on ophthalmologic examination with or without changes in BCVA, observed in 48 of 61 (78.7%) patients in Part 1. Additionally, objective findings of decrease in BCVA assessed by the Snellen method and graded by the prespecified keratopathy and visual acuity (KVA) scale were reported in 83.6% (51 of 61) of patients. Other commonly reported AEs regardless of causality included fatigue in 62.3% (38 of 61), neutropenia in 57.4% (35 of 61), infection in 50.8% (31 of 61) and thrombocytopenia in 52.5% (32 of 61) of patients. The most common grade 3–4 AEs (≥20%) were keratopathy in 57.4% (35 of 61), decreased visual acuity in 49.2% (30 of 61), neutropenia in 45.9% (28 of 61) and thrombocytopenia in 39.3% (24 of 61) of patients.

**Table 2:** Any-grade AEs occurring in ≥20% of patients and grade 3–4 AEs occurring in ≥5% of patients

Any-grade AEs, *n* (%)	Part 1 patients	RP2D patients	All patients
	*n* = 61	*n* = 38	*N* = 87
Keratopathy	48 (78.7)	25 (65.8)	62 (71.3)
Decreased visual acuity (BCVA)	51 (83.6)	27 (71.1)	68 (78.2)
Fatigue	38 (62.3)	22 (57.9)	52 (59.8)
Infection	31 (50.8)	18 (47.4)	44 (50.6)
Neutropenia	35 (57.4)	15 (39.5)	43 (49.4)
Thrombocytopenia	32 (52.5)	15 (39.5)	38 (43.7)
Diarrhea	24 (39.3)	11 (28.9)	30 (34.5)
Fever	22 (36.1)	6 (15.8)	26 (29.9)
Peripheral edema	21 (34.4)	13 (34.2)	28 (32.2)
Constipation	21 (34.4)	11 (28.9)	26 (29.9)

Grade 3–4 AEs, *n* (%)	Part 1 patients	RP2D patients	All patients
	*n* = 61	*n* = 38	*N* = 87
Keratopathy	35 (57.4)	20 (52.6)	48 (55.2)
Decreased visual acuity	30 (49.2)	15 (39.5)	38 (43.7)
Fatigue	9 (14.8)	2 (5.3)	10 (11.5)
Infection	15 (24.6)	3 (7.9)	18 (20.7)
Neutropenia	28 (45.9)	14 (36.8)	36 (41.4)
Thrombocytopenia	24 (39.3)	13 (34.2)	29 (33.3)
Diarrhea	3 (4.9)	3 (7.9)	4 (4.6)

Data on AEs by dosing cohort in Part 1 are in Extended Data Table 2a,b.

**Extended Data Fig. 2:**
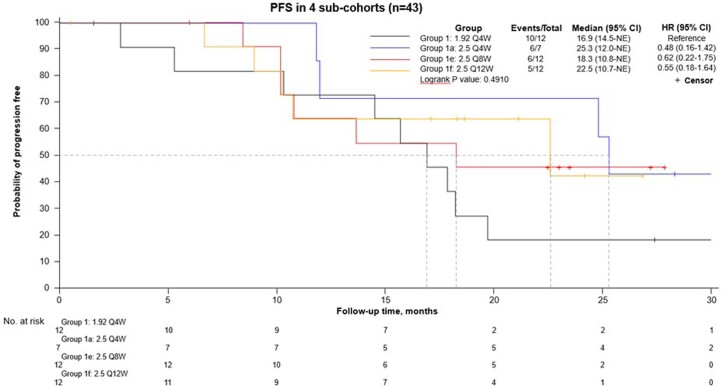
PFS for 1.92mg/kg Q4W and 2.5 mg/kg Q4W, Q8W, and Q12w subgroups. NE, not evaluable; no., number; PFS, progression-free survival.

Although patient numbers for individual groups are small, there was a trend toward lower frequency and less severity of ocular AEs with the 1.92 mg kg^−1^ dose versus the MTD of 2.5 mg kg^−1^ administered on Q4W schedules (Table 3a; 3.4 mg kg^−1^ SPLIT cohort presented in Extended Data Table 3a). Rates of grade 3–4 keratopathy, objective decrease in BCVA by the KVA scale and symptomatic grade ≥2 blurred vision by the National Cancer Institute Common Terminology Criteria for Adverse Events (CTCAE v.5.0) grading were 33.3% (4 of 12), 41.7% (5 of 12) and 25% (3 of 12), respectively, for the 1.92 mg kg^−1^ dose and 100% (7 of 7), 71.4% (5 of 7) and 57.2% (4 of 7), respectively, for the 2.5 mg kg^−1^ Q4W cohort. Since corneal toxicities were managed with dose holds and/or dose reductions for grade 2 keratopathy and grade 2 decrease in BCVA or grade ≥3 keratopathy or decrease in BCVA, the relative dose intensity of belamaf received over the course of the study was lower in the 2.5 mg kg^−1^ Q4W cohort (22%) versus those assigned to the 1.92 mg kg^−1^ dose, in which the relative dose intensity delivered was 88% (Table 3b; 3.4 mg kg^−1^ SPLIT cohort presented in Extended Data Table 3b). Despite the lower actual dose intensity (0.5 mg kg^−1^) delivered over the course of treatment, preliminary efficacy data indicated superior clinical efficacy with the 2.5 mg kg^−1^ initial dose with ORR, ≥very good partial response (VGPR) rate and mPFS of 100% (7 of 7), 100% (7 of 7) and 25.3 months (11.8 to not yet reached (NYR)) versus 66.7% (8 of 11), 63.7% (7 of 11) and 16.9 months (5.3–19.7), respectively, for the 1.92 mg kg^−1^ dose (Extended Data Table 4 and Extended Data Fig. 2). Consequently, in an attempt to preserve efficacy but reduce ocular toxicities, additional dose cohorts exploring the 2.5 mg kg^−1^ dose administered at longer dosing intervals of every 8 weeks (Q8W) and every 12 weeks (Q12W) were included in Part 1. With extended dosing schedules there was a trend toward lower rates of grade 3–4 keratopathy, decreased BCVA and subjective grade ≥2 blurred vision, which were seen in 58.3% (7 of 12), 91.7% (11 of 12) and 16.7% (2 of 12) of patients treated at 2.5 mg kg^−1^ Q8W and 58.3% (7 of 12), 58.3% (7 of 12) and 16.7% (2 of 12) of patients treated at 2.5 mg kg^−1^ Q12W versus the Q4W schedule (Table 3a). Importantly, the efficacy was preserved with ORRs of 91.7% (11 of 12) and 100% (11 of 11), ≥VGPR rates of 83.3% (10 of 12) and 63.7% (7 of 11) and mPFS of 18.3 months (95% confidence interval (95% CI), 10.1 to NYR) and 22.5 months (95% CI, 10.7 to NYR) for the Q8W and Q12W schedules, respectively (Extended Data Table 4 and Extended Data Fig. 2).

**Table 3:** Ocular AEs (Part 1 by cohort and RP2D), summary of belamaf dosing (Part 1 by cohort and RP2D) and summary of pomalidomide dosing (Part 1 by cohort and RP2D)

AEs, maximum grade	1.92 mg kg^−1^ Q4W	2.5 mg kg^−1^ Q4W	2.5 mg kg^−1^ Q8W	2.5 mg kg^−1^ Q12W	2.5 mg kg^−1^ LOADING^a^	2.5 mg kg^−1^ SPLIT	RP2D
	*n* = 12	*n* = 7	*n* = 12	*n* = 12	*n* = 5	*n* = 8	*n* = 38
Keratopathy, grade 2, *n* (%)	2 (16.7)	0 (0.0)	3 (25.0)	1 (8.3)	1 (20.0)	1 (12.5)	4 (10.5)
Keratopathy, grade 3–4, *n* (%)	4 (33.3)	7 (100.0)	7 (58.3)	7 (58.3)	2 (40.0)	5 (62.5)	20 (52.6)
Keratopathy recovery from grade ≥2 to grade 1, *n*/*N* (%)	5/6 (83.3)	5/7 (71.4)	6/10 (60.0)	7/8 (87.5)	1/3 (33.3)	5/6 (83.3)	13/24 (54.2)
Decrease in BCVA, grade 2, *n* (%)	4 (33.3)	2 (28.6)	1 (8.3)	3 (25.0)	1 (20.0)	1 (12.5)	9 (23.7)
Decrease in BCVA, grade 3–4, *n* (%)	5 (41.7)	5 (71.4)	11 (91.7)	7 (58.3)	4 (80.0)	4 (50.0)	21 (55.3)
BCVA recovery from grade ≥2 to grade 1, *n*/*N* (%)	7/9 (77.8)	7/7 (100.0)	4/12 (33.3)	7/10 (70.0)	3/5 (60.0)	5/5 (100.0)	15/30 (50.0)
Blurred vision (patient reported), grade 2, *n* (%)	2 (16.7)	3 (42.9)	0 (0.0)	1 (8.3)	0 (0.0)	1 (12.5)	2 (5.3)
Blurred vision (patient reported), grade 3–4, *n* (%)	1 (8.3)	1 (14.3)	2 (16.7)	1 (8.3)	0 (0.0)	1 (12.5)	5 (13.2)
Other ocular toxicity, grade 2, *n* (%)	1 (8.3)	1 (14.3)	0 (0.0)	1 (8.3)	1 (20.0)	2 (25.0)	4 (10.5)
Other ocular toxicity, grade 3–4, *n* (%)	0 (0.0)	1 (14.3)	0 (0.0)	1 (8.3)	0 (0.0)	0 (0.0)	0 (0.0)

Belamaf dosing	1.92 mg kg^−1^ SINGLE	2.5 mg kg^−1^ Q4W	2.5 mg kg^−1^ Q8W	2.5 mg kg^−1^ Q12W	2.5 mg kg^−1^ LOADING^a^	2.5 mg kg^−1^ SPLIT	RP2D
	*n* = 12	*n* = 7	*n* = 12	*n* = 12	*n* = 5	*n* = 8	*n* = 38
No. of cycles administered, median (range)	14 (2–32)	27 (13–36)	19 (7–30)	13 (1–30)	9 (6–37)	23 (3–45)	15 (1–30)
No. of expected doses, median (range)	14 (2–32)	27 (13–36)	10 (4–15)	5 (1–10)	9 (6–37)	23 (3–45)	8 (1–15)
No. of doses administered, median (range)	7 (2–20)	8 (5–14)	6 (2–11)	3 (1–7)	7 (2–25)	9 (2–19)	4 (1–11)
No. of doses missed, median (range)	3 (0–22)	14 (7–28)	4 (0–9)	2 (0–4)	5 (2–27)	15 (1–26)	3 (0–9)
Intended dose intensity (mg kg^−1^ Q4W)	1.92	2.5	1.25	0.83	2.5^a^	2.5	1.25
Actual dose intensity (mg kg^−1^ Q4W), median (range)	1.7 (0.5–1.9)	0.5 (0.5–1.1)	0.5 (0.2–0.9)	0.3 (0.1–0.8)	0.7 (0.5–1.6)	0.6 (0.2–1.7)	0.5 (0.1–1.3)
Relative dose intensity (%), median (range)	88 (29–100)	22 (19–43)	41 (16–75)	36 (15–100)	29 (20–62)	50 (14–100)	37 (11–100)

Pomalidomide dosing	1.92 mg kg^−1^ SINGLE	2.5 mg kg^−1^ Q4W	2.5 mg kg^−1^ Q8W	2.5 mg kg^−1^ Q12W	2.5 mg kg^−1^ LOADING^a^	2.5 mg kg^−1^ SPLIT	RP2D
	*n* = 12	*n* = 7	*n* = 12	*n* = 12	*n* = 5	*n* = 8	*n* = 38
Patients with ≥1 dose reduction, *n* (%)	7 (58.3)	5 (71.4)	6 (50.0)	6 (50.0)	3 (60.0)	5 (62.5)	12 (31.6)
No. of doses missed, median (range)	0 (0–1)	0 (0–3)	0 (0–1)	0 (0–0)	0 (0–1)	0 (0–1)	0 (0–1)
Intended dose intensity (mg Q4W)	84	84	84	84	84	84	84
Actual dose intensity (mg Q4W), median (range)	84.0 (57.0–86.0)	83.3 (52.6–84.7)	84.0 (54.7–84.0)	81.8 (58.4–84.2)	83.4 (75.8–84.0)	83.4 (50.1–84.0)	84.0 (54.7–86.0)
Relative dose intensity (%), median (range)	100 (70–100)	100 (60–100)	100 (70–100)	100 (70–100)	100 (90–100)	100 (60–100)	100 (70–100)

Keratopathy and BCVA graded per the KVA scale; blurred vision and other ocular toxicity are from CTCAE v.5.0.

Data on the 3.4 mg kg^−1^ SPLIT group are in Extended Data Table 3a.

^a^Loading dose is 2.5 mg kg^−1^ Q4W then reduced to 1.92 mg kg^−1^ Q4W.

Data on the 3.4 mg kg^−1^ SPLIT group are in Extended Data Table 3b.

Data on the 3.4 mg kg^−1^ SPLIT group are in Extended Data Table 3c.

An analysis of hematologic toxicities that are commonly associated with pomalidomide use^14^ indicated that the percentage of patients experiencing grade 3–4 neutropenia or thrombocytopenia was similar for the 1.92 mg kg^−1^ Q4W (*n* = 12) (41.7% and 41.7%, respectively) and 2.5 mg kg^−1^ Q4W (*n* = 7) (42.9% and 42.9%, respectively) cohorts (Extended Data Table 2b). Although the requirements for dose reductions of pomalidomide were higher in patients treated Q4W initiated at the 2.5 mg kg^−1^ dose (71.4%) versus those treated with 1.92 mg kg^−1^ (58.3%) (Table 3c; 3.4 mg kg^−1^ SPLIT cohort presented in Extended Data Table 3c), no patient in the former cohort discontinued pomalidomide due to cytopenias. Overall, the data show that the dose intensity across all dosing cohorts is well preserved and that pomalidomide does not negatively impact the toxicity profile of belamaf administered at higher doses.

Based on the 91.7% (11 of 12) ORR, 83.3% (10 of 12) ≥VGPR rate and better tolerability of an extended dosing schedule, the RP2D was set at 2.5 mg kg^−1^ Q8W with 4 mg of Pd.

### Results of the RP2D cohort

As of 14 February 2023, 38 patients had been enrolled in cohorts receiving belamaf at the RP2D. The median age was 71 yr (range, 38–85), and patients had received a median of 3 (range, 1–6) previous lines of therapy (Table 1). Thirty-one of 38 patients (81.6%) were refractory to both lenalidomide and a PI, and 24 (63.2%) had triple-class refractory disease.

After receiving a median of 15 cycles of treatment (range, 1–30), all 38 patients had experienced ≥1 AE, with treatment-related AEs occurring in 30 (78.9%) patients. Two patients of 38 (5.2%) discontinued treatment due to an AE, 1 for grade 4 thrombocytopenia related to both belamaf and pomalidomide and 1 for grade 4 hypoxia attributed to pomalidomide. There were 4 deaths (10.5%), including 2 possibly related to study drug (1 lung infection with multiorgan failure dosed in Part 2 and 1 COVID-19 dosed at the RP2D in Part 1), 1 of unknown cause, and 1 due to medically assisted death. Grade 3–4 AEs were reported in 37 of 38 patients (97.4%). The most common (≥20%) grade 3–4 AEs were keratopathy in 20 (52.6%), decreased BCVA in 15 (39.5%), neutropenia in 14 (36.8%) and thrombocytopenia in 13 (34.2%) patients (Table 2b).

AEs of clinical interest included thrombocytopenia, neutropenia, infection and ocular AEs. At the RP2D, thrombocytopenia of any grade and grade ≥3 was reported in 15 of 38 (39.5%) and 13 of 38 (34.2%) patients, respectively (Table 2a,b). Any-grade neutropenia occurred in 15 of 38 (39.5%) patients. Grade ≥3 events were observed in 14 of 38 (36.8%) patients, while febrile neutropenia was reported in only 6 of 38 (15.8%) patients. Infections of any etiology or grade occurred in 18 of 38 (47.4%) patients, with grade ≥3 events in 3 of 38 (7.9%) patients. Although numbers are small, it does not appear that the extended dosing schedule reduces the rate of grade ≥3 neutropenia, thrombocytopenia or infection compared with the 2.5 mg kg^−1^ Q4W schedule (*n* = 7) (42.9%, 42.9% and 0%, respectively) (Extended Data Table 2b). Keratopathy based on eye examination and objective decrease in BCVA by the Snellen method were reported in 25 of 38 (65.8%) and 27 of 38 (71.1%) patients, respectively. Despite 21 of 38 (55%) patients having documented grade ≥3 decrease in BCVA, few patients reported a maximum of grade ≥2 blurred vision (7 of 38 (18.4%)), and no patients discontinued belamaf for an ocular AE. At the time of data cut-off, 13 of 24 (54.2%) and 15 of 30 (50.0%) patients with objective findings of grade ≥2 keratopathy or decreased BCVA, respectively, had recovered to grade ≥1. Finally, moderate (grade 2) other ocular AEs, including dry eyes, photophobia and eye pain, were reported in 4 of 38 (10.5%) patients, with no cases of grade 3–4 observed. No irreversible loss of complete vision has been reported. The median number of missed doses of belamaf for AEs was 3 (range, 0–9), and 24 (63.2%) patients required a dose reduction to 1.92 mg kg^−1^; the relative dose intensity delivered was 37% (Table 3b).

**Fig. 2:**
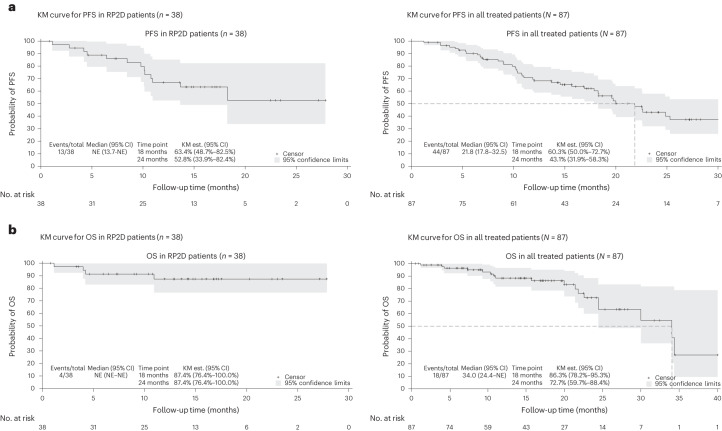
Kaplan–Meier curves for PFS and OS. **a**, PFS in RP2D (*n* = 38) and all treated patients (*N* = 87). **b**, OS in RP2D (*n* = 38) and all treated patients (*N* = 87). Est., estimated; KM, Kaplan–Meier; NE, not evaluable.

**Extended Data Fig. 3:**
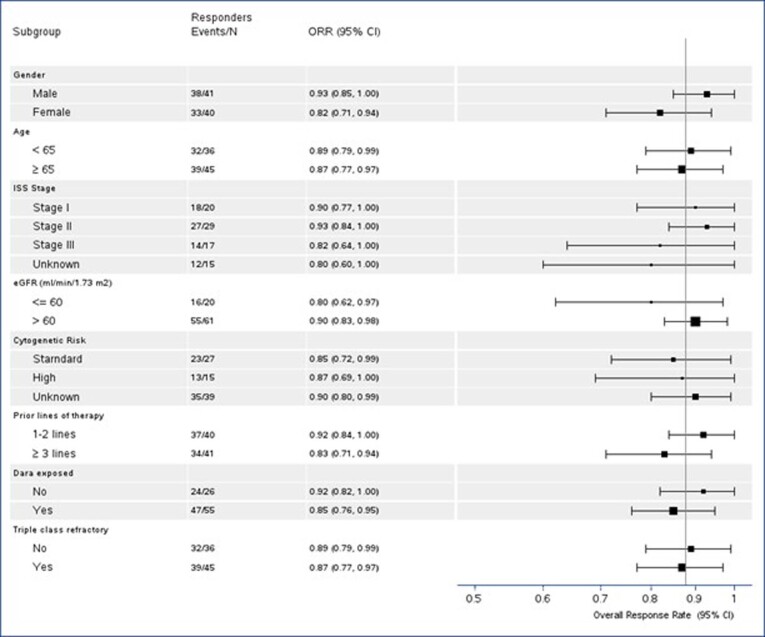
Forest plot for patients with confirmed responses (N = 81) by patient subgroup. The vertical bar for overall response rate (ORR) was set at 0.877 which is the point estimator for the entire study cohort. eGFR, estimated glomerular filtration rate; ISS, International Staging System.

A summary of the efficacy outcomes is presented in Table 4. For patients treated at the RP2D, the ORR based on 2 consecutive assessments was 85.3% (29 of 33), with 75.7% (25 of 33) achieving ≥VGPR and 33.3% (11 of 33) reaching ≥complete response (CR). Seven patients with confirmed CR or better across all dosing cohorts had minimal residual disease (MRD) assessment performed by multiparameter flow cytometry with sensitivity of 10^−5^. Notably, 5 achieved MRD negativity, including 3 of 4 patients treated at the RP2D. With a median follow-up of 13.9 months (range, 1.1–28.2), the mPFS was NYR (range, 13.7 months to NYR), with an estimated 52.8% (95% CI, 33.9% to 82.4%) of patients remaining without disease progression at 2 yr (Fig. 2a). The median overall survival (mOS) has not been met for patients treated at the RP2D, with 87.4% (95% CI, 76.4% to 100%) estimated to be alive at 2 yr (Fig. 2b). Finally, a retrospective exploratory subgroup analysis revealed that the ORR was consistent across all subgroups, including the high-risk cytogenetics and triple-class refractory patients (Extended Data Fig. 3).

**Table 4:** Summary of efficacy data for RP2D and all treated patients (ORR, PFS, OS)

Efficacy outcomes	Part 1	RP2D	All
	*n* = 61	*n* = 38	*N* = 87
ORR, *n*/*N* (%)	53/59 (89.8)	29/34 (85.3)	71/81 (87.7)
CR/sCR, *n*/*N* (%)	20/59 (33.9)	11/34 (33.3)	27/81 (33.3)
VGPR, *n*/*N* (%)	24/59 (40.7)	14/34 (42.4)	32/81 (39.5)
PR, *n*/*N* (%)	9/59 (15.3)	4/34 (11.8)	12/81 (14.8)
mPFS, months (95% CI)	20.0 (15.7–30.0)	NYR (13.7 to NYR)	21.8 (17.8- 32.5)
mOS, months (95% CI)	34.0 (24.0 to NYR)	NYR (NYR to NYR)	34.0 (24.4 to NYR)
Median follow-up, months (range)	17.1 (0.9–42.5)	13.9 (1.1–28.2)	14.5 (0.9–42.5)

sCR, stringent complete response; PR, partial response.

ORR, CR/sCR, VGPR, PR and disease progression were based on two consecutive response assessments. mPFS and mOS were based on intent to treat. Data on individual cohorts from Part 1 are in Extended Data Table 4.

## Discussion

The flexible design of this dose-escalation two-part study allowed the evaluation of multiple doses and administration schedules to better define the optimal balance of anti-MM effect and tolerability when belamaf is combined with full doses of Pd for the treatment of RRMM. Accordingly, this trial established the RP2D dose of belamaf as 2.5 mg kg^−1^ Q8W, which demonstrated promising clinical efficacy with an acceptable safety profile when added to Pd.

The therapeutic landscape of MM has evolved considerably in the last several years, with most patients now being treated with various triplet combinations of IMiDs, PIs and, most recently, anti-CD38 mAbs in the first line or initial relapses based on many studies that consistently indicate the impressive efficacy of these regimens^15–17^. More recently, quadruplets including a PI, IMiD, anti-CD38 mAb and dexamethasone have entered the newly diagnosed space, showing even more remarkable activity^18,19^. Due to these new treatment standards, in jurisdictions where patients can access these therapies, the vast majority will be exposed and potentially refractory to all three major classes of drug after first- or second-line therapy; therefore, there is an urgent need for novel drug targets and combinations beyond those currently available in early relapses of MM.

BCMA is a novel therapeutic target in the current era, with belamaf the first anti-BCMA antibody–drug conjugate in clinical use. Despite promising single-agent activity^2^, the randomized phase 3 DREAMM-3 trial comparing single-agent belamaf with Pd did not meet its primary endpoint^20^. Although the mPFS was longer for the belamaf arm, 11.2 months versus 7.0 months for Pd, this did not meet statistical significance (hazard ratio 1.03; 95% CI, 0.72 to 1.47). Evaluation of the Kaplan–Meier curves demonstrated crossover of the progression-free survival (PFS) curves at the 3–4-month mark as a result of early disease progression. Notably, however, the PFS for the belamaf arm stayed stably superior to that of Pd after the crossover. Consistently, the mDOR for belamaf was not reached at the time of the analysis, with clear separation of the curves in favor of belamaf. It has been postulated that the durability of response observed in the DREAMM studies reflects the ability of belamaf to induce immunogenic cell death^1^. It seems logical, therefore, that incorporating belamaf in combination with other active anti-MM agents may induce more rapid disease control to mitigate early progression while extending the clear DOR benefit. Indeed, the results of the ALGONQUIN study demonstrate a doubling of responses and a corresponding improvement of PFS.

The results of DREAMM-3 also reaffirm the findings that Pd is active as a doublet combination, but the DOR is limited^3^. To improve outcomes, many pomalidomide-based triplet regimens have been studied. The most efficacious appear to be combinations with the anti-CD38 mAbs. Pd with daratumumab was evaluated in a phase 2 study, MM-014 (ref. ^21^), and the randomized phase 3 APOLLO trial^10^. MM-014 was limited to patients with 1 or 2 previous therapies and lenalidomide exposure, and it produced a mPFS of 30.8 months and 23.7 months for those patients refractory to lenalidomide^21^. In the APOLLO trial of patients with ≥1 previous line (median, 2) and both lenalidomide and PI exposure, those randomized to the daratumumab-Pd arm had a mPFS of 12.4 months (ref. ^10^). The combination of pomalidomide and isatuximab has been evaluated in the phase 3 ICARIA trial of patients with ≥2 previous treatment lines including lenalidomide and a PI, in which those randomized to the isatuximab-Pd arm had a mPFS of 11.1 months (ref. ^22^).

However, the utility of these pomalidomide–anti-CD38 combinations will mostly be limited to selected MM patients going forward due to the earlier use of anti-CD38 mAbs as mentioned. Pd has also been evaluated in several other non-anti-CD38 mAb triplet combinations in relapsed MM. These include Pd in combination with elotuzumab (ELOQUENT-3)^23^, bortezomib (OPTIMISMM)^24^, selinexor (STOMP)^25^ and cyclophosphamide^26^, demonstrating mPFS results ranging from 9.5 to 12.2 months (refs. ^23–26^). These findings highlight the need for more effective treatments with newer targets and combination regimens. In the present study, in which patients had received a median of 3 previous lines of therapy and 66.7% of patients were anti-CD38 exposed, the ORR of the overall study population was 87.6%, with a mPFS of 21.8 months (range, 17.8–24.2) and a mOS of 34.0 months (range, 30.0 to NYR). For patients treated at the RP2D of belamaf 2.5 mg kg^−1^ Q8W with Pd, the ORR was 84.8% and mPFS was NYR (range, 13.7 months to NYR), with an estimated 2-yr PFS and OS of 52.8% (range, 32.9–83.4%) and 87.4%, respectively, at median follow-up of 13.9 months. These results represent an improvement on the efficacy demonstrated in studies of the other aforementioned pomalidomide-based combinations, with an approximate doubling of the PFS.

The other agents currently in clinical use for patients who are triple-class exposed are the anti-BCMA bispecific mAbs (bispecifics) and the anti-BCMA chimeric antigen receptor (CAR) T cell therapies. Several bispecific antibodies have shown efficacy in relapsed MM, including elranatamab^27^ and teclistamab^28^, which are approved by both the US Food and Drug Administration and the European Medicines Agency. In the phase 2 MagnetisMM-3 trial of single-agent elranatamab, an ORR of 61% and estimated mPFS of 50.9% at 15 months were reported^29^. Patients treated with teclistamab in the MajesTEC-1 trial were heavily pretreated with a median of 5 previous lines of treatment and achieved a similar ORR of 63% and an mPFS of 12.5 months (ref. ^30^). While the ORR for teclistamab was improved when combined with lenalidomide and daratumumab in patients having received only 2 previous lines of therapy at 93.5%, the incidence of infections was 90.6%, suggesting that this combination strategy may be problematic^31^. From an efficacy perspective, recognizing the limitations of cross-trial comparisons, our response rates and durability appear to be competitive with the anti-BCMA bispecifics currently available.

In addition, there are some distinct challenges with toxicity associated with the bispecifics, in particular cytokine release syndrome (CRS) and infections. In the MagnetisMM-3 trial, CRS was reported in 50.6% of patients (although all grade 1–2), and 61.8% had infections, of which 31.7% were grade 3–4 (ref. ^27^). Patients treated in the MajesTEC-1 study also had high rates of low-grade CRS (72%), and high rates of infection (76% overall, with 44.8% grade 3–4)^28^. In the present study, the risk of serious infection was low in comparison, with 47.4% of patients having infection of any grade and 7.9% experiencing grade 3–4 infection at the RP2D. These results are also favorable when compared with the rates of grade 3–4 infections reported for the combinations of anti-CD38 mAbs and Pd (23% in the APOLLO study^10^ and 22.8% respiratory infections and pneumonia in the ICARIA study^21^). There were, however, 6 of 87 (6.9%) deaths on study due to infection, including 3 secondary to COVID-19. In addition, atypical opportunistic infections, including pneumocystis pneumonia and progressive multifocal leukoencephalopathy, likely attributable to the combination with dexamethasone and pomalidomide, were reported. Thus, the use of growth factor support, infection prophylaxis and immunoglobulin replacement therapy for immunoparesis should be considered with this regimen.

CAR T cell therapy is an active option for some patients with relapsed MM, with both idecabtagene vicleucel (ide-cel)^32^ and ciltacabtagene autoleucel (cilta-cel)^33^ approved and available in some jurisdictions. In the KARMMA-3 study single-agent ide-cel produced an mPFS of only 13.3 months in patients who similarly had received a median of 3 lines of therapy and of whom 65% were triple-class refractory^32^. On the other hand, the efficacy seen in the CARTITUDE-1 patients who ultimately received cilta-cel is unprecedented in this space, with an mPFS of 33.9 months in patients with a median of 6 previous lines of therapy^34^. Despite these encouraging efficacy results, access to CAR T cell therapy in relapsed MM is currently challenging due to production capacity, manufacturing failures as well as the rapidity of progression for many patients who are unable to wait the length of time needed for CAR T cell manufacturing. Indeed, in the recently published phase 3 study of cilta-cel in early relapsed MM, 15.4% of patients did not receive CAR T cell infusion mostly due to disease progression, resulting in an ORR of 84.6% in the intent-to-treat population^35^. In these situations, an ‘off the shelf’ option may be preferred, in which case belamaf plus Pd represents a highly effective combination.

Overall, the safety profile of the belamaf-Pd combination was consistent with that of the individual agents. The main clinical challenge, however, is undoubtedly belamaf-related ocular toxicity. In our study, 65.8% of patients treated at the RP2D had alterations in the cornea on ophthalmologic examination and 71.1% experienced changes in BCVA. However, only 18.4% reported subjective symptoms of blurred vision grade ≥2 by CTCAE grading, and no patients discontinued belamaf due to ocular symptoms. Regular monitoring and examinations with an eye care professional are essential, which does contribute to the overall encumbrance of treatment for patients. In addition, clinicians must be knowledgeable in interpreting ocular examination results as well as managing ocular toxicity with respect to both symptom awareness and belamaf dose modifications. In our study, we found less ocular toxicity in the 1.92 mg kg^−1^ cohort versus the 2.5 mg kg^−1^ dose given Q4W, which resulted in 100% of patients experiencing grade 3–4 keratopathy. However, preliminary efficacy data favored the 2.5 mg kg^−1^ dose. After studying the 2.5 mg kg^−1^ dose at extended dosing intervals (Q8W and Q12W), which resulted in a similar actual dose intensity versus the Q4W interval due to fewer dose holds, we were able to reduce the burden of grade 3–4 ocular toxicity without compromising clinical efficacy. Despite the improvement in the safety profile, the actual dose delivered was only 37% of the intended dose, and thus there remains a challenge with consistent administration of belamaf at the 2.5 mg kg^−1^ dose even on a Q8W schedule.

Limitations of this study include the small sample size for determination of the optimal tolerable dose and schedule of belamaf in each cohort, and the need for further confirmatory studies on this topic. Our conclusions, however, are supported by recently presented data in newly diagnosed patients similarly showing that belamaf Q12W plus lenalidomide and dexamethasone reduced the frequency of grade 3 BCVA changes from baseline to 11–13% (ref. ^36^). Thus, longer dosing intervals may represent a important step forward in mitigating the corneal toxicities of belamaf. Further, the single-arm design of the study poses uncertainty as to the clinical benefits of belamaf-Pd compared with other available treatments for patients with relapsed MM. The phase 3 DREAMM-8 study will provide further data on the mPFS of belamaf-Pd in comparison with the standard of care regimen of bortezomib plus Pd.

In summary, the ALGONQUIN trial demonstrated that the combination of belamaf plus Pd resulted in promising efficacy for patients with relapsed MM, comparable to the anti-BCMA bispecific antibodies and ide-cel CAR T cell therapy and an improvement over other Pd-based combinations. Moderate and severe ocular symptoms occurred less with an extended dosing schedule without compromising efficacy. These results support belamaf-Pd as a BCMA-directed option for the management of relapsed MM.

## Methods

### Study design, patients and conduct

The ALGONQUIN study is an ongoing, multicenter, open-label, single-arm, two-part study (ClinicalTrials.gov Identifier: NCT03715478) of belamaf plus Pd in patients with RRMM. Part 1 of the study consisted of a dose-exploration phase. A standard 3 + 3 dose escalation was used, with up to 6 patients enrolled at doses of 1.92, 2.5 and 3.4 mg kg^−1^ belamaf in combination with Pd to determine the MTD (Extended Data Fig. 1). The option of evaluating additional cohorts exploring alternative dosing schedules at the MTD or lower and/or of enrolling up to 12 patients per cohort to better inform the RP2D and schedule was included in the protocol. The dose-exploration phase was followed by an expansion cohort in Part 2 to evaluate the safety, tolerability and clinical activity of the dose and schedule identified in Part 1.

At screening, patients with RRMM were eligible if they were aged ≥18 yr; had an Eastern Cooperative Oncology Group performance status of 0–2; had undergone autologous stem cell transplantation or were considered transplant ineligible; had experienced disease progression after ≥1 previous lines of anti-myeloma treatment and must have been lenalidomide refractory and PI exposed (in separate regimens or in combination); and had adequate bone marrow, renal and cardiac function. Patients with previous pomalidomide or BCMA-target therapy exposure, concurrent corneal epithelial disease (except mild punctate keratopathy), any serious and/or unstable preexisting medical condition, a psychiatric disorder or any other condition (including laboratory abnormalities) that could interfere with the patient’s safety, obtaining informed consent or compliance with the study procedures were excluded. Sex was not considered in the study design or patient selection and was determined based on self-reporting.

The study was conducted at nine Canadian sites in accordance with the Declaration of Helsinki and International Council Harmonisation Good Clinical Practices Guidelines. The study protocol, amendments and informed consent were approved by the institutional review boards at each participating site. The study complied with local regulation governing the conduct of clinical studies and institutional guidelines. All patients provided written, informed consent. The data were collected by the sponsor and all authors had full access and were involved in data interpretation, manuscript preparation, revision and final approval. The authors vouch for the accuracy of the data and adherence to the study protocol.

### Procedures and study endpoints

Belamaf was administered as a 30-min intravenous infusion every 4 weeks at a dose of 1.92 mg kg^−1^ (cohort 1); or every 4, 8 or 12 weeks at a dose of 2.5 mg kg^−1^ (cohorts 1a, 3a and 3b); or at total doses of 2.5 or 3.4 mg kg^−1^ Q4W but split (SPLIT) evenly with 50% of the dose administered on days 1 and 8 of every cycle (cohorts 1b and 2, respectively); or as a loading dose of 2.5 mg kg^−1^ on cycle 1 day 1, followed by a dose reduction to 1.92 mg kg^−1^ from cycle 2 onward on a Q4W schedule (cohort 1c). Pomalidomide was administered at 4 mg on day 21 of 28 and dexamethasone 40 mg (20 mg if aged >75 yr) weekly. Patients remained on treatment until disease progression, unacceptable toxicity or consent withdrawal. Ophthalmology examinations before each dose of belamaf and preservative-free lubricant eye drops were required throughout study treatment. Dose modifications were made independently for each drug according to predefined criteria based on the nature and toxicity grade of the event.

### Assessments

AEs were assessed for severity using CTCAE v.5.0, with the exception of visual acuity by the Snellen method and corneal epithelium changes observed on ophthalmic examination, which were graded by the prespecified KVA scale^37^. Clinical activity of the combination was assessed by the treating physician in accordance with the International Myeloma Working Group Uniform Response Criteria for Multiple Myeloma^38^.

### Endpoints

The primary endpoints for Part 1 of the study were to determine the MTD, RP2D and schedule of belamaf when given in combination with Pd. Dose-limiting toxicities occurring during the first 28-d treatment cycle were used to determine the MTD. The dose for Part 2 expansion was chosen based on review of the totality of the safety, tolerability and activity data during Part 1. An additional primary endpoint of the study for patients treated at the RP2D in Parts 1 and 2 was to determine the ORR (partial response or better) for all response-evaluable patients, defined as those with two consecutive response assessments. The secondary endpoints were to determine PFS, OS and safety assessments in all treated patients. An additional planned secondary endpoint not reported in this manuscript is DOR. Exploratory objectives included pharmacokinetic profiles, rate of MRD negativity, PFS2, pharmacodynamic changes in immune cells, and molecular alterations present in myeloma cells and their response to selective pressures of treatment. These will be reported separately.

### Statistical analysis

No formal statistical power calculations were performed to determine the sample sizes for the dose-exploration portion of the study. All analyses of outcomes from Part 1 of the study were descriptive, with results reported by dose and schedule, as relevant. For the RP2D cohort a sample size of 35 was obtained to detect a response rate of 0.60 against 0.30 as the historical response rate^2,3^, a one-side binomial proportional test was performed, type I error rate was set at 0.05 and the power was set at 97%. Also, a 10% drop rate was considered. Two-sided 95% (Clopper–Pearson) CI was calculated for ORR in the dose escalation and all patients treated at the RP2D. All patients who had ≥1 dose of study drug were included in the analyses. Data are summarized by all treated patients, dose group and RP2D-dosed patients. PFS and OS were analyzed using the Kaplan–Meier method. Number of patients at risk and event-free rates (with 95% CI) at specific months were displayed within the survival curves. The SAS v.9.4 (SAS Institute) software package was used for analyses.

### Reporting summary

Further information on research design is available in the Nature Portfolio Reporting Summary linked to this article.

## Online content

Any methods, additional references, Nature Portfolio reporting summaries, source data, extended data, supplementary information, acknowledgements, peer review information; details of author contributions and competing interests; and statements of data and code availability are available at 10.1038/s41591-023-02703-y.

### Supplementary information

Reporting Summary

## Figures and Tables

**Extended Data Table 1:**
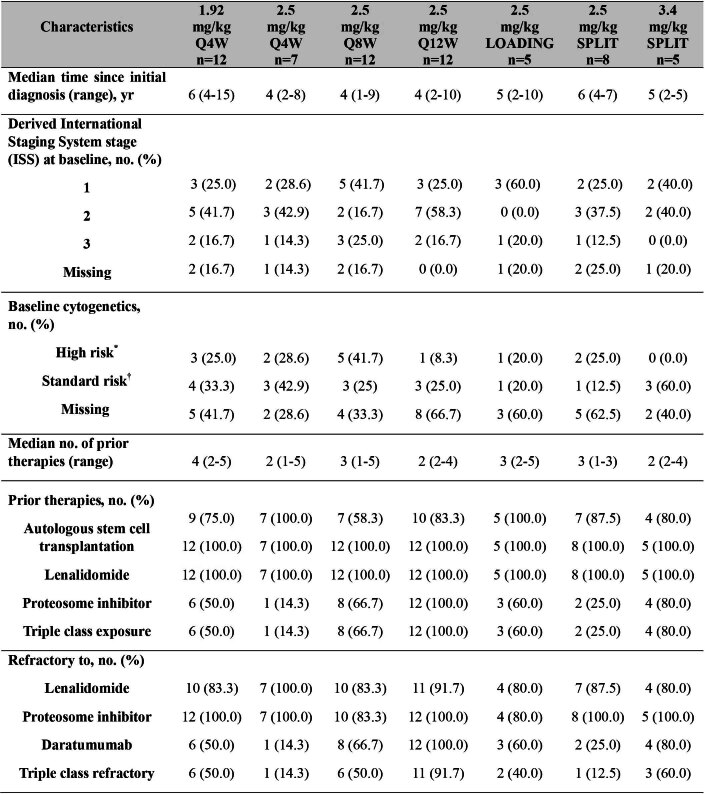
Patient Characteristics From Part 1 by Dosing Cohort

No., number; yr, years. *High risk is defined as patients presenting with abnormality for del(17p) and/or translocation t(4;14) and/or translocation t(14;16). Fluorescence in situ hybridization was performed locally using the individual laboratory’s cut-off values. ^†^Standard risk is defined as patients with absence of abnormality for all of the following: del(17p), translocation t(4;14), and translocation t(14;16). Dose-escalation patients include patients from cohorts 1, 1a, 1b, 1c, 1e, 1f and 2 (Fig. 1).

**Extended Data Table 2:**
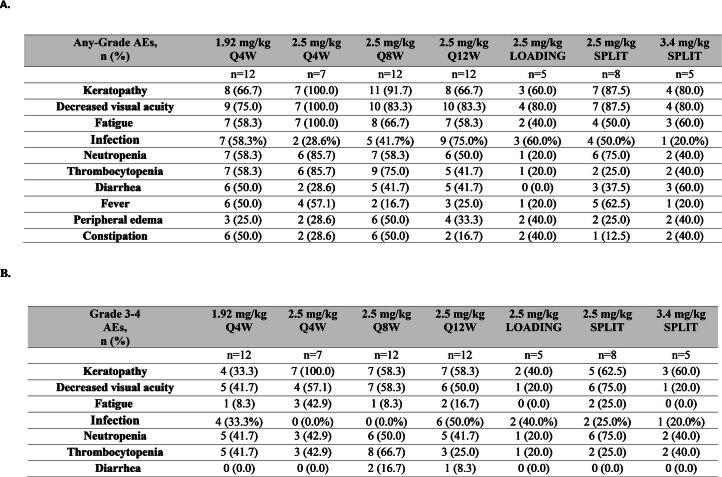
A. Any-Grade AEs Occurring in ≥20% of Patients by Dosing Cohort, Part 1; B. Grade 3-4 AEs Occurring in ≥5% of Patients by Dosing Cohort, Part 1

AE, adverse event.

**Extended Data Table 3:**
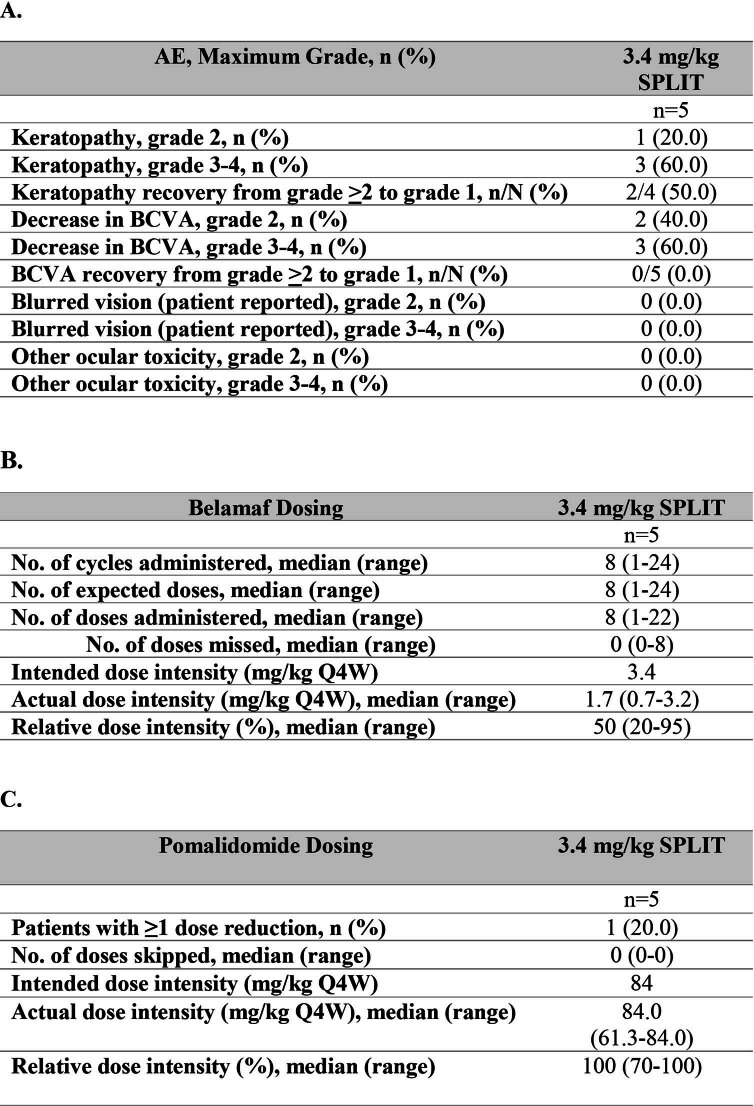
A. Ocular AEs in the 3.4 mg/kg SPLIT Dose Cohort; B. Belamaf Dosing in the 3.4 mg/kg SPLIT Dose Cohort; C. Pomalidomide Dosing in the 3.4 mg/kg SPLIT Dose Cohort

AE, adverse event; BCVA, best corrected visual acuity; No., number.

**Extended Data Table 4:**
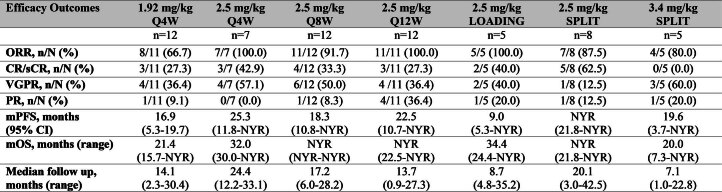
Efficacy Data for Part 1 by Dosing Cohort

CR, complete response; mOS, median overall survival; mPFS, median progression-free survival; NYR, not yet reached; ORR, overall response rate; PR, partial response; sCR, stringent complete response; VGPR, very good partial response.

## Data Availability

Upon request, and subject to review, participant consent and local privacy laws, the Canadian Myeloma Research Group (CMRG) will provide access to data that support the findings of this study. For data requests, please contact the CMRG at contact@cmrg.ca. Please allow up to 2 weeks for a response.

## Code availability

Not applicable; coding was not used.
